# The Herbst Method of Filling with Glass

**Published:** 1890-11

**Authors:** James A. Bruce

**Affiliations:** Melbourne, Australia


### THE HERBST METHOD OF FILLING WITH GLASS.

BY JAMES A. BRUCE, D.D.S., MELBOURNE, AUSTRALIA.

Having seen an article on Dr. Herbst's glass fillings in one of the dental journals, and regarding it as rather indefinite as to the process, I propose to give a more detailed account. It was my good fortune to spend a short time with Dr. Herbst, so that I feel I can, possibly, make the subject clear to those who may wish to try it.

There must be three or four kinds, or rather colors, of glass,—viz., white, brown, and yellow, with occasionally a little blue,—so as to adapt the filling to the color of the tooth. The best results obtained by Dr. Herbst are produced by using beads, careful to obtain those having very little or no transparency, such as milk-glass. These are ground very fine and mixed according to the color of your tooth. I find the best way is to have a good average color, and for any alteration necessary a small quantity of white, yellow, or brown, as the case may need.

Prepare the cavity in the mouth without any undercuts; but leaving it with nice, sharp edges, the latter being an important part in the success of the filling.

Next, line the cavity with one layer of No. 60 gold foil, pressing it to place with india-rubber or some such material, and finally taking a wax impression (the wax being used hard), the gold being withdrawn in the wax. The result is a sharp impression of the cavity into which plaster and pumice, in proportion of two to one, respectively, is run. After this is thoroughly set, boil out the wax, leaving your model with the gold coating.

Proceed with the glass filling by placing a few grains of sand in the bottom of the cavity (to make the cement adhere firmly) and a little glass mixture, moistened on the top, very little more than enough to hold the sand together. Dry thoroughly, and then make use of a Bunsen burner and mouth blow-pipe, the former being necessary to get rid of the smoke. Heat until a blaze is formed, then allow it to cool off, and then add your second coating, which very nearly fills the cavity, and proceed as before. The third coating is to bring up the glass exactly to the edge of the cavity. It is done in the same way as the others and forms the last layer. This process very much resembles continuous-gum work; but the excessive heat is not needed. When cool, remove the filling from the plaster and gold and it is ready for insertion in the mouth.

Before inserting, however, make slight undercuts in the cavity without altering the outer edge. The gold used gives a very much sharper edge to the glass filling, and altogether seems to fit the cavity better than when doing without it.

The filling is made in a very few moments and can be prepared by an assistant, and in places, such as the labial surfaces, can with practice be made a very good match to the color of the natural tooth and a perfect fit. Perhaps I might add that it is always well to have the filling slightly darker than lighter than the tooth. These fillings when once inserted into cavities with cement are so firm that it is almost impossible to remove them.

Dr. Herbst adds to the above the following suggestions:

"It is somewhat difficult to procure the right kind of beads. They are such as are used for needle-work. There should be one color mixed with three parts of the white and one part of the brown glass ground previously in an agate mortar as fine as possible. First try the color by burning one layer and add a little of the white or brown glass as the filling may require."

